# Activity of irinotecan and temozolomide in the presence of O^6^-methylguanine-DNA methyltransferase inhibition in neuroblastoma pre-clinical models

**DOI:** 10.1038/sj.bjc.6605927

**Published:** 2010-10-05

**Authors:** W Cai, N V Maldonado, W Cui, N Harutyunyan, L Ji, R Sposto, C P Reynolds, N Keshelava

**Affiliations:** 1 Division of Hematology-Oncology, MS#119, Childrens Hospital Los Angeles, 4650 Sunset Boulevard, Los Angeles, CA 90027, USA; 2 Department of Pediatrics, University of Southern California, Los Angeles, CA 90027, USA; 3 Department of Preventive Medicine, Keck School of Medicine, University of Southern California, Los Angeles, CA 90027, USA; 4 Department of Pathology, University of Southern California, Los Angeles, CA 90027, USA

**Keywords:** active drug combinations, cytotoxicity, drug resistance, recurrent neuroblastoma

## Abstract

**Background:**

The combination of temozolomide (TMZ) and irinotecan is a regimen used in neuroblastoma patients with recurrent disease. O^6^-methylguanine-DNA methyltransferase (MGMT) may have a function in resistance to TMZ. Using neuroblastoma pre-clinical models, we determined whether the inhibition of MGMT by O^6^-benzylguanine (O6-BG) could enhance the anti-tumour activity of TMZ and irinotecan.

**Methods:**

The cytotoxicity of TMZ and irinotecan, either alone or in combination, was measured in five neuroblastoma cell lines in the presence or absence of O6-BG with a fluorescence-based cell viability assay (DIMSCAN). Anti-tumour activity was measured in three neuroblastoma xenograft models.

**Results:**

MGMT mRNA and protein were expressed in 9 out of 10 examined cell lines. Pretreatment of cells with 25 *μ*M O6-BG decreased MGMT protein expression and enhanced The TMZ cytotoxicity by up to 0.3–1.4 logs in four out of five tested cell lines. TMZ (25 mg kg^−1^ per day for 5 days every 3 weeks for four cycles) did not significantly improve mice survival, whereas the same schedule of irinotecan (7.5 mg kg^−1^ per day) significantly improved survival (*P*<0.0001) in all three xenograft models. Combining O6-BG and/or TMZ with irinotecan further enhanced survival.

**Conclusion:**

Our *in vitro* and *in vivo* findings suggest that irinotecan drives the activity of irinotecan and TMZ in recurrent neuroblastoma. Inhibitors of MGMT warrant further investigation for enhancing the activity of regimens that include TMZ.

Neuroblastoma is the most common extracranial solid tumour of childhood. Myeloablative chemotherapy followed by hematopoietic stem-cell transplantation and then 13-*cis* retinoic acid (Matthay *et al*, 1999) or most recently anti-GD2 antibody and cytokines (Yu *et al*, 2009) improve survival of children with high-risk neuroblastoma, but many patients still die of progressive disease. Therefore, the development of new therapies is a priority.

Irinotecan, a topoisomerase I inhibitor, is a semi-synthetic water-soluble analogue of camptothecin, a pro-drug that undergoes de-esterification to an active metabolite, SN-38. Irinotecan, as a single agent, has shown activity *in vitro* (Keshelava *et al*, 2000) and in xenograft neuroblastoma models (Vassal *et al*, 1996; Thompson *et al*, 1997; Furman *et al*, 1999), and demonstrated some objective responses in neuroblastoma patients treated with various doses and schedules (Furman *et al*, 1999; Blaney *et al*, 2001; Kushner *et al*, 2005; Bomgaars *et al*, 2006, 2007). Preclinical data from rhabdomyosarcoma and neuroblastoma xenograft models have demonstrated schedule-dependent activity for irinotecan, with superior activity seen when using protracted low-dose administration (Houghton *et al*, 1995).

Temozolomide (TMZ), a DNA methylating imidazole tetrazinone, inhibits cell growth in neuroblastoma xenografts (Middlemas *et al*, 2000) and has been shown to have activity in high-risk neuroblastoma patients (De *et al*, 2006; Rubie *et al*, 2006). It has been hypothesised that the cytotoxic activity of TMZ is mediated through reactive O^6^-methylguanine in DNA (Tisdale, 1987); thus, inhibition of O^6^-methylguanine-DNA methyltransferase (MGMT), a DNA repair protein at the O^6^-guanine position, may increase the cytotoxicity of TMZ against neuroblastomas (Wagner *et al*, 2007b). O^6^-benzylguanine (O6-BG) is a potent suppressor of MGMT activity, acting as a substrate for MGMT (Dolan *et al*, 1990).

Irinotecan is commonly used in combination with TMZ to treat recurrent high-risk neuroblastomas (Wagner *et al*, 2009) because of their synergistic interaction in xenograft models (Houghton *et al*, 2000), and their activity in patients with Ewing sarcoma (Wagner *et al*, 2007a) and high-grade glioma (Reardon *et al*, 2005). Clinically, these agents are administered sequentially, starting with TMZ, because of the schedule-dependent activity demonstrated in xenograft models. It is presumed that the placement of an adduct at the O^6^ position of guanine by an alkylating agent, such as BCNU or TMZ, is critical for achieving synergistic activity with irinotecan (Castellino *et al*, 2000).

The purpose of the current study was to assess the activity of the TMZ and irinotecan combination in neuroblastoma pre-clinical models and to determine whether inhibition of MGMT by O6-BG significantly enhanced the anti-tumour activity of the combination.

## Materials and methods

### Drugs and chemicals

Both TMZ and O6-BG were obtained from the Drug Synthesis and Chemistry Branch, Developmental Therapeutics Program, National Cancer Institute (Bethesda, MD, USA). SN-38 was purchased from Abatra Technology Co., Ltd, and irinotecan was purchased from the pharmacy at Childrens Hospital Los Angeles. Fluorescein diacetate was purchased from Eastman Kodak Company (Rochester, NY, USA), and eosin Y was from Sigma Chemical Co. (St Louis, MO, USA). Antibodies were purchased from various manufacturers: anti-MGMT monoclonal MT 3.1 Ab-1 from NeoMarkers (Fremont, CA, USA), caspase-3 (Cat# 9665) and phospho-H2AX (Ser 139, Cat# 9718) rabbit monoclonal antibody from Cell Signaling Technology (Danvers, MA, USA) and F7-26 from Chemicon International (Temecula, CA, USA).

### Cell Lines

We used four drug-sensitive (SMS-SAN, CHLA-42, CHLA-15 and SMS-KCNR) and seven multidrug-resistant (CHLA-171, CHLA-136, SK-N-RA, SK-N-BE(2), CHLA-90, CHLA-119 and CHLA-172) cell lines. CHLA-136 and SK-N-RA cell lines carry wild type and transcriptionally active *TP53* (Keshelava *et al*, 2001). SK-N-BE(2), CHLA-90, CHLA-119 and CHLA-172 carry mutant and transcriptionally inactive *TP53* (Keshelava *et al*, 2001). CHLA-171 carries wild type, but transcriptionally inactive *TP53*, likely because of the MDM2 protein overexpression (Keshelava *et al*, 2001).

SMS-KCNR cells were grown in RPMI-1640 supplemented with 10% heat-inactivated fetal bovine serum (Omega Scientific, Tarzana, CA, USA). All other cells were grown in Iscove's Modified Dulbecco's Medium (Biowhittaker, Walkersville, MD, USA) supplemented with 20% fetal bovine serum, 3 mM L-Glutamine (Mediatech, Inc., Herndon, VA, USA) and 0.1% premium ITS culture supplement containing 5 *μ*g ml^−1^ insulin, 5 *μ*g ml^−1^ transferrin and 5 ng ml^−1^ selenious acid (VWR Scientific, West Chester, PA, USA). The cell lines were cultured without antibiotics at 37°C in a humidified incubator containing 95% air and 5% CO_2_ atmosphere. All cell lines tested negative for mycoplasma. Cell lines were not selected for drug resistance *in vitro*. The identities of all cell lines were confirmed by a short tandem repeat assay (Masters *et al*, 2001).

### TaqMan RT-PCR

TaqMan real-time reverse transcription-PCR (RT-PCR) was used to quantify RNA with the Sequence Detection System (ABI Prism model 7700; Applied Biosystems, Foster City, CA, USA). The probe and primer for MGMT gene were purchased from Applied Biosystems (Cat# Hs00172470).

Reactions were performed with 150 ng of RNA in the Master Mix prepared from the kit, TaqMan one-step RT-PCR Master Mix Reagents (Applied Biosystems, Branchburg, NJ, USA), in a final volume of 25 *μ*l. Three replicates for each reaction were plated into 96-well plates. RT-PCR assay was performed under the following conditions: 2 min at 50°C, 10 min at 95°C, 40 cycles of denaturation at 95°C for 15 s and annealing/extension at 60°C for 1 min. The RNA of each sample was normalised to the level of GAPDH.

### Cytotoxicity assay

We determined the cytotoxicity of O6-BG, irinotecan and TMZ, as single drugs or in combination, in the CHLA-15, CHLA-42, SMS-KCNR, CHLA-136 and CHLA-90 cell lines by DIMSCAN (Keshelava *et al*, 2005; Frgala *et al*, 2007), a semiautomatic fluorescence-based digital image microscopy system that quantifies viable cells in tissue culture multi-well plates on the basis of their selective accumulation of fluorescein diacetate. DIMSCAN is capable of measuring cytotoxicity over a four-log dynamic range by quantifying total fluorescence per well, which is proportional to the number of viable, clonogenic cells after eliminating background fluorescence with digital thresholding and eosin Y quenching (Keshelava *et al*, 2005; Frgala *et al*, 2007).

Briefly, cell lines were seeded into 96-well plates in 150 *μ*l of complete medium (1500–3500 cells per well) and incubated overnight. O6-BG (25 *μ*M) in 50 *μ*l of complete medium was added to each well. After an, additional, 24-h incubation, TMZ (0–50 *μ*g ml^−1^) and SN-38, an active *in vitro* metabolite of irinotecan (0–20 ng ml^−1^), were added. We chose a range of concentrations for TMZ and SN-38 that would encompass drug levels active in pre-clinical models and clinically relevant. Protracted administration of low-dose irinotecan in children resulted in a *C*_max_ of ∼20 ng ml^−1^ (Ma *et al*, 2000), a level that was cytotoxic to neuroblastoma cells (Keshelava *et al*, 2000). The clinically achievable level of TMZ in children is 13±2.8 *μ*g ml^−1^ (Horton *et al*, 2007) and in xenograft models, TMZ *C*_max_ is 36 *μ*g ml^−1^ (Houghton *et al*, 2000). Each drug concentration was tested in 12 replicate wells. Cell lines were incubated in the presence of these agents for 72 h, after which fluorescein diacetate in 50 *μ*l of 0.5% eosin Y (final concentration of fluorescein diacetate=10 *μ*g ml^−1^) was added to each well and cells were incubated for an additional 25 min at 37°C. Total fluorescence was then measured with the use of DIMSCAN as previously described (Keshelava *et al*, 2005; Frgala *et al*, 2007), and results were expressed as surviving fractions of treated cells compared with control cells that were exposed to vehicle solution alone.

In addition to simultaneous administration of TMZ and SN-38, we also tested sequential administration of these drugs in the CHLA-15, SMS-KCNR and CHLA-90 cell lines. In these experiments, TMZ was added to cells 3 h before irinotecan.

### ssDNA damage measurements with F7-26 antibody

Cells were treated with O6-BG (25 *μ*M, 24 h), TMZ (50 *μ*g ml^−1^, 6 h), SN-38 (20 ng ml^−1^, 4 h) or their combinations, and analysed after overnight fixation in methanol–PBS (6:1). Staining with F7-26 monoclonal antibody was performed according to the manufacturer's instructions. Flow cytometry was performed on a BD LSR II flow cytometer (BD Biosciences, San Jose, CA, USA) and data were acquired and analysed using BD FACSDiva software.

### Immunoblot analysis of protein expression

Cells from three drug-sensitive cell lines (SMS-KCNR, SMS-SAN and CHLA-42) and seven multidrug-resistant cell lines (CHLA-171, CHLA-136, SK-N-RA, SK-N-BE(2), CHLA-90, CHLA-119 and CHLA-172) were grown to 70% confluence in 75 cm^2^ flasks and then lysed in immunoprecipitation assay buffer (50 mM NaCl, 50 mM Tris (pH 7.4), 1% Triton X-100, 1% sodium deoxycholate, 0.1% sodium dodecyl sulphate). Total protein (30 *μ*g per lane) was fractionated on 10% tris-glycine precast gels (Novex, San Diego, CA, USA), transferred to nitrocellulose membrane (Protran, Keene, NH, USA), and probed with primary antibodies at a 1 : 1000 dilution, followed by incubation with an HRP-conjugated secondary antibody. The membranes were incubated with SuperSignal West Pico chemiluminescent substrate (Pierce Biotechnology, Inc., Rockford, IL, USA), and protein: antibody complexes were detected by autoradiography (Denville Scientific, Inc., Metuchen, NJ, USA).

H2AX expression, phosphorylated at Ser 139, was assessed in CHLA-15 cells for characterisation of DNA double strand breaks. Similar doses and schedules were tested as with F7-26 monoclonal antibody staining.

### Animals

Athymic (nu/nu) female mice, 6–7 weeks old (Harlan Laboratories, Inc., San Diego, CA, USA) were used in the study. Mice were housed in an air-conditioned room and fed the standard laboratory chow. All procedures were performed in compliance with the Childrens Hospital Los Angeles Institutional Animal Care and Use Committee-approved protocols.

### Mouse models of mass disease

We used the SMS-KCNR, CHLA-136 and CHLA-119 neuroblastoma cell lines for the xenograft experiments. These cell lines were chosen to represent neuroblastomas that were: (a) drug-sensitive (SMS-KCNR), (b) multidrug-resistant with wild-type and transcriptionally active *TP53* (CHLA-136) and (c) multidrug-resistant with mutant and transcriptionally inactive *TP53* (CHLA-119) (Keshelava *et al*, 2001). Treatments began when tumours reached 200 mm^3^. Tumour measurements were made twice a week. Once tumour reached 1500 mm^3^, mice were humanely sacrificed.

### Treatment protocol

Drugs were given as follows: O6-BG (30 mg kg^−1^ delivered through intraperitoneal injections) was followed 1 h later by TMZ (25 mg kg^−1^ given by oral gavage) and 1 h later by irinotecan (7.5 mg kg^−1^ given by tail vein injection). O6-BG was dissolved in 4% DMSO, 30% PEG-400 and 66% saline, TMZ was dissolved in water, and irinotecan in saline. Control mice were administered sterile carrier solutions. Mice were treated on a 1-week on (5 days)-2 weeks off schedule for four cycles. The doses of administered drugs were chosen following determination of the maximum tolerated dose. The administered drugs at the doses reported here were well tolerated; no weight loss, diarrhea or abnormal behaviour was observed. White blood cell count was not monitored.

### Data analysis

IC_90_ (drug concentrations that were cytotoxic or growth inhibitory for 90% of the cell population), and dose reduction index values were calculated using CalcuSyn software (Biosoft, Cambridge, UK) (Chou and Talalay, 1984).

The association between log-transformed RT-PCR values for MGMT RNA expression and immunoblot values for MGMT protein expression was assessed by calculating the Pearson's correlation coefficient. All *P*-values are two-sided. The computations of the Pearson's correlation coefficient were performed with the use of STATA software (version 9.0; College Station, TX, USA). A *P*-value of less than or equal to 0.05 was considered statistically significant.

The effect of TMZ pre-treatment was studied by comparing dose–response curves of the TMZ and irinotecan combination, in the presence or absence of O6-BG, with those drugs administered simultaneously or sequentially. The difference in the cytotoxic effect was examined by comparing the area between the dose–response curve and the 100% survival line. Statistical analysis assumed that the difference between the untreated controls and the highest drug concentration was 1. The fixed ratio design of the cytotoxicity experiments allowed the concentration increments to be quantitated at 1/8, 1/4, 1/2 and 1. The area between the survival curves for each treatment group and the 100% survival line was viewed as the sum of areas of four trapezoids, computed as: 
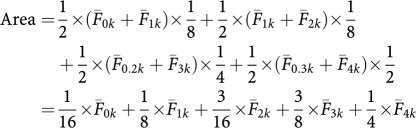
 where *F*_*jk*_ is the mean fraction of logs of cell kill after treatment at the *j*th dose (0th, 1st, 2nd, 3rd and 4th drug conditions). The linear combination: 
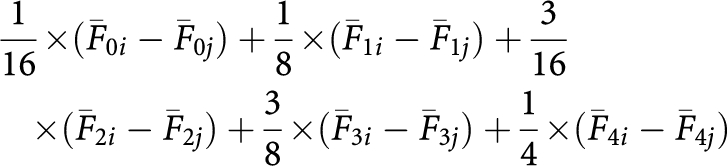
 then tested the difference in treatment effects between two dose–response curves (designated as *i* and *j*). Data were analysed with software SAS Version 9.1 (College Station, TX, USA).

For xenograft studies, the main effects of the three drugs (O6-BG, TMZ and irinotecan) on the length of survival of mice, as well as their second- and third-order interactions (e.g., enhancement of the effect of one drug with addition of another) were evaluated using censored regression models as implemented in the CNREG routine of STATA 9.0 (StataCorp LP, College Station, TX, USA). Days of survival were assumed to have a lognormal distribution, and the days were transformed to the natural log scale before analyses were carried out. O6-BG, TMZ and irinotecan, as well as their second- and third-order interaction terms were included in the initial regression model. For main drug effects, a *P*-value less than or equal to 0.05 was considered statistically significant. For test of interaction effect between drugs (e.g., enhancement in the effect of one drug with addition of another), a *P*-value less than or equal to 0.01 was considered statistically significant. *P*-values from likelihood ratio tests are presented. To compare the length of survival between any two groups of mice, a censored regression model was first fit using data from all the treatment groups with the CNREG routine, then difference in mice survival between two treatment groups was evaluated by testing the appropriate linear combinations using the STATA LINCOM command. Data from the three cell lines was analysed separately. All *P*-values reported were two-sided.

## Results

### MGMT RNA and protein expression

**Figure 1:**
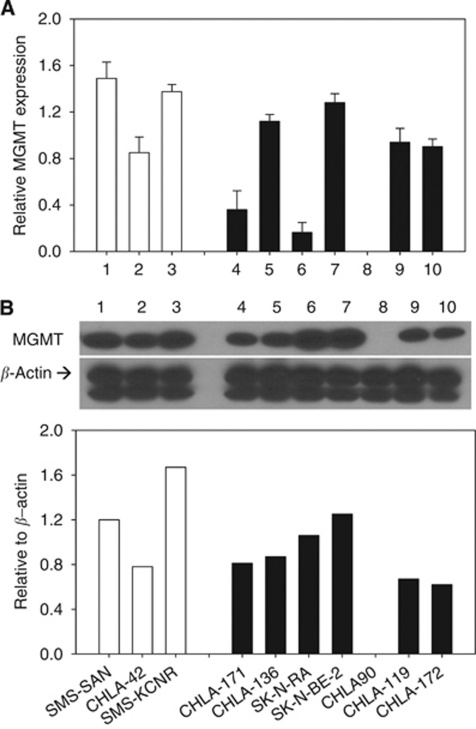
O^6^-methylguanine-DNA methyltransferase (MGMT) expression in drug-sensitive (white bars) and multidrug-resistant neuroblastoma cell lines (black bars). Lanes, 1 – SMS-SAN, 2 – CHLA-42, 3 – SMS-KCNR, 4 – CHLA-171, 5 – CHLA-136, 6 – SK-N-RA, 7 – SK-N-BE(2), 8 – CHLA-90, 9 – CHLA-119 and 10 – CHLA-172. (**A**) Quantification of mRNA expression for the MGMT gene by TaqMan reverse transcription–polymerase chain reaction in a panel of 10 neuroblastoma cell lines. Mean mRNA expression (normalised to GAPDH mRNA expression) and standard deviations (error bars) from three replicates are shown. (**B**) Upper panel: Immunoblot analysis of MGMT protein expression, equal protein loading was confirmed by immunoblot analysis of *β*-actin expression. Bottom panel: quantitative analysis of MGMT protein expression as a ratio to *β*-actin expression.

Around 10 neuroblastoma cell lines (SMS-SAN, SMS-KCNR, CHLA-42, CHLA-171, CHLA-136, SK-N-RA, SK-N-BE(2), CHLA-90, CHLA-119 and CHLA-172) were tested for MGMT RNA and protein expression by TaqMan RT-PCR and immunoblotting, respectively (Figure 1A and B). All cell lines, except CHLA-90, expressed MGMT. MGMT RNA expression correlated significantly with MGMT protein expression (*r*=0.67, *P*<0.03). Thus, MGMT is expressed in neuroblastomas and may be a pharmacological target for therapeutic regimens that include TMZ, as previously suggested (Wagner *et al*, 2007b).

### Cytotoxicity of O6-BG, TMZ and irinotecan in neuroblastoma cell lines

**Figure 2:**
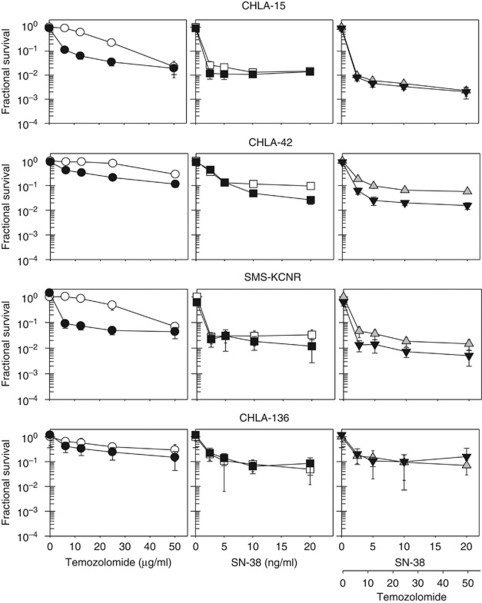
Effect of the temozolomide and SN-38 combination in the presence of O^6^-methylguanine-DNA methyltransferase (MGMT) inhibitor in neuroblastoma cell lines obtained by DIMSCAN assay. For MGMT inhibition, cells were pretreated with 25 *μ*M O^6^-benzylguanine (O6-BG) for 24 h, temozolomide and/or SN-38 were then added for an additional 3 days. Dose–response curves for temozolomide alone (*○*), temozolomide in the presence of O6-BG (•), SN-38 alone (□), SN-38 in the presence of O6-BG (▪), temozolomide and SN-38 (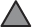) and temozolomide and SN-38 in the presence of O6-BG (▾) were obtained by DIMSCAN assay.

**Figure 3:**
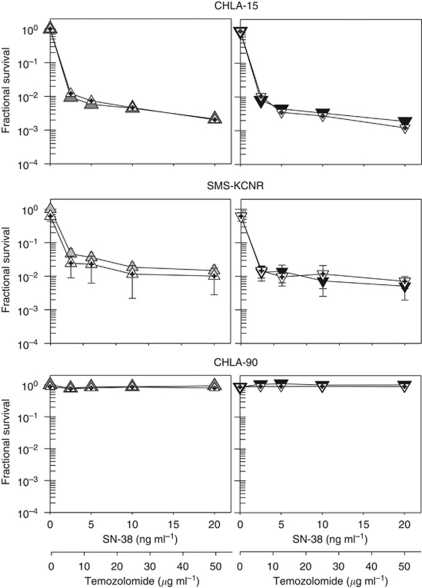
Cytotoxicity of sequentially or simultaneously administered temozolomide and SN-38 in neuroblastoma cell lines measured by the DIMSCAN assay. Cells were pretreated with 25 *μ*M O^6^-benzylguanine (O6-BG) for 24 h, temozolomide and SN-38 were then added for an additional 3 days. Temozolomide was added 3 h before or simultaneously with SN-38. Dose–response curves for temozolomide and SN-38 administered simultaneously (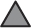) or sequentially (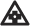), temozolomide and SN-38 administered simultaneously in the presence of O6-BG (▾), temozolomide+SN-38 administered sequentially in the presence of O6-BG (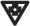).

Next, we examined whether O6-BG can increase the activity of TMZ or the TMZ and irinotecan combination in neuroblastomas using three drug-sensitive (CHLA-15, CHLA-42 and SMS-KCNR) and two multidrug-resistant (CHLA-90 and CHLA-136) cell lines, including the one that does not express MGMT (CHLA-90) (Figures 2 and 3). For these *in vitro* experiments, O6-BG was tested at 25 *μ*M, a concentration chosen on the basis of the studies demonstrating significant reduction of MGMT enzymatic activity *in vitro* by O6-BG at 10 *μ*M (Bacolod *et al*, 2002). Depletion of MGMT protein by 75 *μ*M O6-BG has also been demonstrated (Murakami *et al*, 2007). Using western blotting, we confirmed reduced protein expression following treatment with 25 *μ*M O6-BG in the SMS-KCNR and CHLA-136 cell lines (data not shown).

Figure 2 shows the dose–response curves obtained from the DIMSCAN assay. We calculated the concentrations that were growth inhibitory or cytotoxic for 90% of treated cells (IC_90_) for single drugs or drug combinations using the dose–response curves for TMZ, SN-38 and TMZ and SN-38 in the presence or absence of 25 *μ*M O6-BG.

The single-agent IC_90_ values for TMZ ranged from 27 to >50 *μ*g ml^−1^ (Table 1). Addition of 25 *μ*M O6-BG enhanced the growth inhibitory effect of TMZ by up to 1.4 logs in CHLA-15, 0.6 logs in CHLA-42, 0.9 logs in SMS-KCNR and 0.3 logs in CHLA-136 cells (Figure 2). The single-agent IC_90_ values for SN-38 ranged from <2.5 to >20 ng ml^−1^ (Table 1). Addition of 25 *μ*M O6-BG marginally enhanced growth inhibitory effect of SN-38 in the CHLA-42 cell line. The TMZ and SN-38 anti-neuroblastoma activity was tested at a fixed ratio of 2.5 : 1. The growth inhibitory activity of the combination was SN-38 driven in all tested lines. Addition of 25 *μ*M O6-BG increased TMZ and SN-38-mediated growth inhibition by up to 0.6 logs in CHLA-42 and 0.5 logs in SMS-KCNR cell lines (Figure 2).

As TMZ is given before irinotecan to neuroblastoma patients, we examined whether administration of TMZ 3 h before SN-38 exposure would result in higher cytotoxicity in representative neuroblastoma cell lines: CHLA-15, SMS-KCNR and CHLA-90. Comparison of the dose–response curves of TMZ and SN-38 in the presence or absence of O6-BG, with the drugs administered simultaneously or sequentially, did not reach statistical significance (Figure 3).

### DNA damage *in vitro*

**Figure 4:**
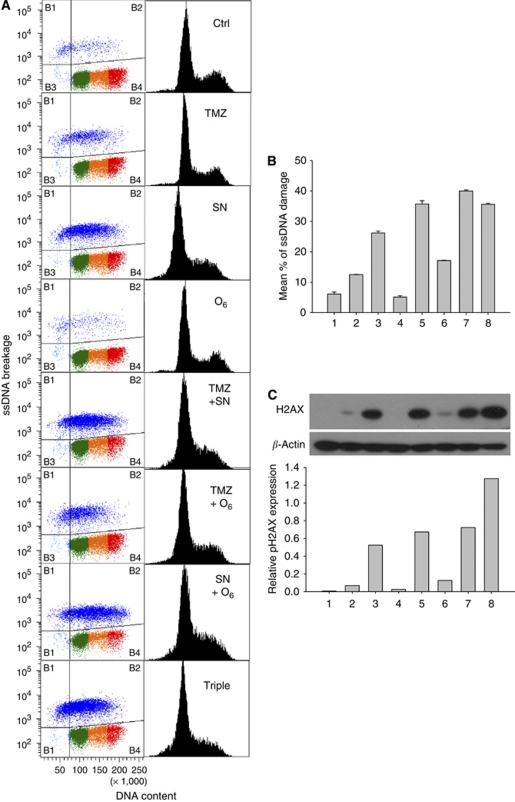
Assessment of single- and double-stranded DNA damage by O^6^-benzylguanine (O6-BG), temozolomide, irinotecan and their combinations in a representative neuroblastoma cell line. CHLA-15 cells were treated with O6-BG (25 *μ*M, 24 h), temozolomide (TMZ, 50 *μ*g ml^−1^, 6 h), and SN-38 (20 ng ml^−1^, 4 h). (**A**) Single-strand breaks were quantified by F7-26 monoclonal antibody using flow cytometric assay. Each condition was examined as three replicates. (**B**) Quantification of single-strand DNA breaks as measured by flow cytometry. Mean values and standard deviation were calculated from three replicates. (**C**) The marker of double-strand DNA-damage, H2AX phosphorylated at Ser 139 (15 kDA), was measured by immunoblotting. Equal protein loading was confirmed by immunoblot analysis of *β*-actin expression. Upper panel: immunoblot analysis of pH2AX expression. Bottom panel: quantitative analysis of pH2AX protein expression as a ratio to *β*-actin expression. Lanes: 1 – untreated controls, 2 – TMZ, 3 – SN-38, 4 – O6-BG, 5 – TMZ and SN-38, 6 – TMZ and O6-BG, 7 – SN-38 and O6-BG and 8 – Triple.

Single- or double-strand (ss or ds) DNA breaks caused by the tested drugs and drug combinations were measured in a representative neuroblastoma cell line (CHLA-15), selected for its propensity to grow in single-cell suspension that does not clump excessively. F7-26 monoclonal antibody staining to single-stranded DNA was used to quantify ssDNA damage (Figure 4A and B). We have previously shown that this method distinguishes ssDNA breaks caused by exogenous stimuli from DNA strand breaks resulting from apoptosis (Grigoryan *et al*, 2007). Phosphorylation of the H2A histone variant, H2AX, at serine-139 was used to quantify dsDNA damage (Figure 4C).

SN-38 induced more prominent single-strand (4.3-fold higher F7-26 staining) or double-strand (84-fold higher expression of pS^139^-H2AX) DNA damage, compared with TMZ (ssDNA=two-fold, dsDNA=11-fold) or O6-BG (ssDNA=1 and dsDNA=4). Significantly more ssDNA damage was achieved by any of the two drug combinations (O6-BG and TMZ, O6-BG+SN-38 and TMZ and SN-38, *P*-values <0.001) relative to simple addition of single drug actions as measured by linear regression analysis. Synergistic induction of ssDNA breaks was not demonstrated for the triple drug combination. Thus, the data show that increased DNA damage by the two- and three-drug combinations results mostly from the breakage induced by SN-38, although TMZ and/or O6-BG further enhanced DNA breaks.

### Activity in the mouse xenograft models

**Figure 5:**
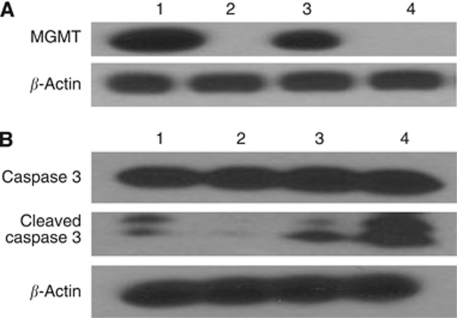
Assessment of pharmacodynamic markers of O^6^-benzylguanine (O6-BG) and temozolomide activity. (**A**) SMS-KCNR cells were established as subcutaneous (s.c.) xenografts in nu/nu mice. Expression of O^6^-methylguanine-DNA methyltransferase was determined in tumour tissue following 5 days of administration of O6-BG at 30 mg kg^−1^ each day. (**B**) Pro-caspase 3 (35 kDa) and cleaved caspase 3 (19 and 17 kDa) were measured by immunoblotting in tumour tissues of SMS-KCNR xenografts after daily administration of O6-BG and/or temozolomide (25 mg kg^−1^) for 5 days. Lanes: 1 – controls, 2 – mice treated with O6-BG, 3 – mice treated with temozolomide, 4 – mice treated with the combination of O-6BG and temozolomide. Equal protein loading was confirmed by immunoblot analysis of *β*-actin expression.

First, we confirmed that the planned dosing schedule achieved the desired pharmacodynamic effect on the xenograft tumours. SMS-KCNR mice were treated with O6-BG or/and TMZ for 1 week and sacrificed. Tumour tissues were obtained and MGMT depletion by O6-BG was demonstrated by immunoblotting (Figure 5A), whereas induction of apoptosis was verified by cleavage of caspase 3 (Figure 5B).

Formal factorial design was applied to our xenograft studies for comprehensive assessment of contributions of single drugs or two-drug combinations to the anti-tumour activity obtainable by the O6-BG, TMZ and irinotecan combination (Table 2).

TMZ as a single agent did not have any significant effect on survival of either SMS-KCNR (*P*=0.21), CHLA-136 (*P*=0.81) or CHLA-119 (*P*=0.9) xenografts; however, addition of O6-BG resulted in significant evidence of enhancement of TMZ effect in SMS-KCNR xenografts (*P*=0.002). This enhancement was marginally significant in CHLA-119 (*P*=0.018) xenografts.

O6-BG, as a single agent, significantly improved survival of the CHLA-136 xenografts (*P*=0.007). When administered as single agents, TMZ and O6-BG did not show significant difference in mean survival days in SMS-KCNR (*P*=0.29) or CHLA-119 (*P*=0.13) xenografts, but showed significant difference in CHLA-136 (*P*=0.028).

**Figure 6:**
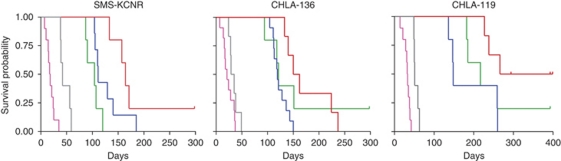
Human neuroblastoma cell lines SMS-KCNR, CHLA-119 and CHLA-136 were established as s.c. xenografts in nu/nu mice. Animals received O^6^-benzylguanine (O6-BG, 30 mg kg^−1^) followed 1 h later by temozolomide (TMZ, 25 mg kg^−1^) and 1 h later by irinotecan (IRN, 7.5 mg kg^−1^). Control mice were administered sterile carrier solutions. Mice were treated for 5 days every 3 weeks for four cycles. Animals were sacrificed when tumours reached 1500 mm^3^. Survival probability of mice treated with vehicle control (pink lines), O6-BG and TMZ (gray lines), IRN (green lines), TMZ and IRN (blue lines), O6-BG, TMZ and IRN (red lines) are shown.

Irinotecan delivered the most activity among the treated groups (Figure 6). As a single agent, irinotecan significantly improved the survival rate (*P*<0.0001) in all three xenograft models. Addition of TMZ (Figure 6) or O6-BG did not significantly improve irinotecan activity. In contrast, addition of O6-BG to the TMZ and irinotecan combination significantly increased survival days of SMS-KCNR (*P*=0.017), CHLA-136 (*P*=0.033) and CHLA-119 (*P*=0.017) xenografts (Figure 6). Notably, 60% of the CHLA-119 xenografts were still alive at 400 days, indicating a significant effect on survival of the O6-BG, TMZ and irinotecan combination.

## Discussion

The combination of TMZ, a DNA methylating agent, with irinotecan, a topoisomerase I inhibitor, had emerged as a promising regimen for recurrent neuroblastoma (Kushner *et al*, 2006; Wagner *et al*, 2009). However, recent evidence suggests that the effectiveness of TMZ in neuroblastomas can be diminished by MGMT-mediated DNA damage repair (Middlemas *et al*, 2000; Wagner *et al*, 2007b). MGMT is frequently expressed in neuroblastoma tumours and cell lines (Middlemas *et al*, 2000; Wagner *et al*, 2007b). MGMT methylation in the promoter region has also been examined in neuroblastomas as promoter hypermethylation is associated with decreased MGMT expression. In those studies, promoter hypermethylation ranged from 0 to 27% in the examined tumours and cell lines (Harada *et al*, 2002; Gonzalez-Gomez *et al*, 2003; Lazcoz *et al*, 2007). Depletion of MGMT activity by the selective inhibitor, O6-BG, reportedly enhanced the cytotoxicity of TMZ in human brain tumour (Bobola *et al*, 1996) and melanoma (Wedge *et al*, 1997) xenografts. O6-BG also enhanced the anti-tumour activity of the TMZ and irinotecan combination in a model of disseminated neuroblastoma (Wagner *et al*, 2007b) and glioma xenograft models (Friedman *et al*, 2002).

We undertook this large-scale study to formally assess the anti-tumour activities of O6-BG, TMZ and irinotecan, as well as their second- and third-order interactions (such as enhancement of one-drug effect with addition of another) in neuroblastoma pre-clinical models. To our knowledge, such extensive assessment has not been carried out previously.

In our study, 10 neuroblastoma cell lines were examined for MGMT RNA and protein expression. All expressed MGMT, except the multidrug-resistant cell line, CHLA-90, which carries a *TP53* mutation (Keshelava *et al*, 2001). This is consistent with a report showing that promoter methylation of the MGMT gene (which results in low or absent MGMT expression) is associated with an increased frequency of *TP53* mutations (Nakamura *et al*, 2001).

We have evaluated the anti-neuroblastoma activity of O6-BG, TMZ and irinotecan *in vitro* and *in vivo*. Drug levels used for O6-BG decreased MGMT protein expression both *in vitro* (data not shown) and *in vivo* (Figure 5A). Our *in vitro* data confirmed that O6-BG enhanced the cell sensitivity to TMZ. Pre-treatment with 25 *μ*M O6-BG sensitised four out of the five tested neuroblastoma cell lines to TMZ by 0.3–1.4 logs (Figure 2); sensitising effect was not demonstrated in the highly drug-resistant CHLA-90 cells that lack MGMT expression. In xenografts, O6-BG as a single agent had a significant effect on the survival of CHLA-136 tumour-bearing mice (*P*=0.007). O6-BG significantly enhanced TMZ activity in SMS-KCNR (*P*=0.002) and marginally in CHLA-119 (*P*=0.018) mice.

Temozolomide, either alone (Rubie *et al*, 2006) or in combination with irinotecan (Wagner *et al*, 2009), is being actively investigated as a possible first-line regimen for recurrent neuroblastomas. In our study, single agent TMZ did not have a significant effect on the survival of either SMS-KCNR, CHLA-136 or CHLA-119 xenografts at the administered dose and schedule. We carefully selected the drug levels for our *in vitro* (0–50 *μ*g ml^−1^) and xenograft experiments (25 mg kg^−1^ per day), on the basis of the previously reported pre-clinical and clinical pharmacokinetic data of TMZ. The pharmacokinetic studies by Stevens *et al*, and Houghton *et al*, conducted in mice, demonstrated that oral administration of 20 mg kg^−1^ or 66 mg kg^−1^ temozolomide resulted in a *C*_max_ of 19.6 *μ*g ml^−1^ (Stevens *et al*, 1987) or 36 *μ*g ml^−1^ (Houghton *et al*, 2000), respectively. The TMZ *C*_max_ levels achievable in children are in the range of 5.6±1.8 *μ*g ml^−1^ (Baruchel *et al*, 2006) to 13±2.8 *μ*g ml^−1^ (Horton *et al*, 2007). Thus, administration of 25 mg kg^−1^ per day TMZ in mice would result in a systemic exposure well within the clinically relevant drug levels. By demonstrating caspase 3 activation, a hallmark of apoptosis (Figure 5B), in SMS-KCNR xenografts, we confirmed the suitability of the administered drug dose.

Irinotecan, as a single-agent, had substantial activity *in vitro* in four neuroblastoma cell lines, achieving 1.1–2.8-log cytotoxicity at 20 ng ml^−1^ (achievable in patients, Vassal *et al*, 2003), and in our xenograft models. Our results are in agreement with other pre-clinical investigations of irinotecan that demonstrated activity against neuroblastomas (Komuro *et al*, 1994; Vassal *et al*, 1996; Thompson *et al*, 1997; Furman *et al*, 1999). However, phase II clinical trials of single-agent irinotecan have only shown limited activity in neuroblastoma patients (Bomgaars *et al*, 2007; Vassal *et al*, 2008). Such divergence could be attributed to the disparate metabolism of irinotecan into its active metabolite SN-38 in different species. It is estimated that mouse liver and kidney carboxylesterase more actively hydrolyses irinotecan than either human carboxylesterase1 (hCE1) (173 *vs* 2.5 pmol mg^−1^ min^−1^ activity) (Xie *et al*, 2003) or hCE2, a more potent activator of irinotecan than human carboxylesterase1 (Humerickhouse *et al*, 2000). Nonetheless, the strong anti-neuroblastoma activity achieved by irinotecan in all our three xenograft models warrants further testing of irinotecan with additional agents to identify synergistic combinations against recurrent neuroblastomas.

In contrast to previous pre-clinical investigations of TMZ and irinotecan in neuroblastoma xenograft models (Houghton *et al*, 2000), our studies did not demonstrate synergy between these drugs. The difference may be attributable to the use in the studies by Houghton *et al* of dose levels of irinotecan and TMZ that were unlikely to induce complete responses when administered alone. In our study, standardised dose-schedule for irinotecan and TMZ were used to mimic the clinical application of O6-BG, TMZ and irinotecan in children. In addition, the 5-day schedule for irinotecan instead of the (dx5(x2)) schedule used by Houghton *et al* may have also contributed to the different responses seen in these two pre-clinical studies.

Previous studies have examined the effect of O6-BG on the anti-tumour activity of the TMZ and irinotecan combination in neuroblastomas (Wagner *et al*, 2007b) and in gliomas (Friedman *et al*, 2002; Quinn *et al*, 2009). A recent phase I clinical trial in adults with recurrent malignant glioma evaluated the combination of O6-BG, TMZ and irinotecan, administered once on 21-day schedule (Quinn *et al*, 2009). Although the primary goal of the study was to define the maximum tolerated dose and dose-limiting toxicity of the evaluated drugs, it did not demonstrate any responses. The lack of responses was hypothesized to be due to subtherapeutic doses or timing of administered doses. Our *in vivo* studies, conducted in a mouse model of mass disease, demonstrated significantly improved survival for xenografts treated with the TMZ and irinotecan combination in the presence of O6-BG, in agreement with the findings by Wagner *et al* in a mouse model of metastatic disease (Wagner *et al*, 2007b). Our data suggest that the enhanced anti-neuroblastoma activity by the triple combination resulted from significant irinotecan activity together with the added benefit of statistically significant O6-BG (in CHLA-136) or O6-BG and TMZ (in SMS-KCNR and CHLA-119) activities. This is confirmed by the DNA studies where single- or double-stranded DNA damage was mostly due to SN-38 and further enhanced with the addition of TMZ and/or O6-BG, emphasising the critical cytotoxic effect of irinotecan in the combination. Moreover, we observed improved survival at 400 days of the multidrug-resistant xenograft CHLA-119 in contrast to the study by Wagner *et al*, in which the apparent advantage of adding O6-BG to TMZ and irinotecan seen at 100 days was lost at 300 days. The difference between these two studies can be explained by the use of four cycles of therapy in our study rather than two as employed by Wagner *et al*, or it may be attributable to the utilisation of the different strain of mice.

Our extensive *in vitro* and *in vivo* investigations using formal factorial design allowed us to convincingly demonstrate in neuroblastoma pre-clinical models that (a) irinotecan administered on a protracted schedule has remarkable anti-tumour activity; (b) MGMT inhibition enhances the anti-tumour activity of TMZ, confirming MGMT as a relevant pharmacological target. These data suggest that inhibition of MGMT should be further investigated especially in regimens containing TMZ and identification of new drug combinations for irinotecan are warranted for the treatment of high-risk neuroblastomas.

## Figures and Tables

**Table 1:** IC values for TMZ and SN-38, and DRI values for their combination in the absence or presence of O6-BG at the 90% growth inhibition

					**DRI^a^**		**DRI_O6-BG_ b**	
**Cell line**	**IC_90_ TMZ (*μ*g ml^−1^)**	**+O6-BG^c^**	**IC_90_ SN-38 (ng ml^−1^)**	**+O6-BG**	**TMZ (+SN38)**	**SN-38 (+TMZ)**	**TMZ (+SN38)**	**SN-38 (+TMZ)**
CHLA-15	31.2	7.2	<2.5	<2.5	>5.0	ND^d^	ND	ND
CHLA-42	>50^e^	>50	14	7.2	>3.3	2.3	>8	>2.9
SMS-KCNR	27.2	<6.25	<2.5	<2.5	>4.3	ND	ND	ND
CHLA-136	>50	>50	6.7	9.3	>2	0.7	>6.8	0.9
CHLA-90	>50	>50	>20	>20	ND	ND	ND	ND

Abbreviations: DRI=dose reduction index; IC_90_=drug concentrations that were cytotoxic or growth inhibitory for 90% of the cell population; ND=not determined; O^6^-BG=O^6^-benzylguanine; TMZ=temozolomide.

a DRI reflects the fold reduction in the required concentration of tested agents when used in combination to achieve 90% cell death and/or growth inhibition.

b DRI for drugs determined in the presence of 25 *μ*M O6-BG.

c IC_90_ values for temozolomide or SN-38 calculated in the presence of 25 *μ*M O6-BG.

d DRI was not determined as over 90% cell death and/or growth inhibition was achieved by a single drug (SN-38 or TMZ) at the concentration that was lower (<2.5 ng ml^−1^ or <6.25 *μ*g ml^−1^) than that tested. DRI was also not determined when 90% cell death and/or growth inhibition was not achieved by drug combination.

e If 90% cell death and/or growth inhibition was not achieved at the tested concentrations, then the IC_90_ values were reported as ‘> the highest-tested concentration’. The highest concentrations tested were: 50 *μ*g ml^−1^ for temozolomide and 20 ng ml^−1^ for SN-38. Similarly, if 90% cell death and/or growth inhibition was achieved at the concentrations lower than that tested, then the IC_90_ values were reported as ‘< the lowest-tested concentration’. The lowest concentrations tested were: 6.25 *μ*g ml^−1^ for temozolomide and 2.5 ng ml^−1^ for SN-38.

**Table 2:** Survival of neuroblastoma xenografts in response to TMZ and/or IRN in the presence or absence of O6-BG

	**SMS-KCNR**			**CHLA-136**			**CHLA-119**		
**Treatment**	***N* a**	**Mean days^b^**	**(95% CI^c^)**	***N***	**Mean days**	**(95% CI)**	***N***	**Mean days**	**(95% CI)**
Control	10^d^	16.6	(11.9, 23.3)	11	20.7	(14.9, 28.8)	10	29.7	(23.3, 37.3)
O6-BG	5	19.3	(13.2, 27.9)	6	32.8	(25, 42.9)	5	25.0	(18, 34.8)
TMZ	5	16.0	(11.4, 22.4)	5	21.3	(13.6, 33.1)	5	37.7	(10.9, 131.6)
IRN	5	100.5	(85.6, 119.1)	5	146.9	(86.6, 249.1)	5	239.2	(166.3, 344.2)
O6-BG+TMZ	5	45.6	(35.2, 59.7)	6	27.7	(18.4, 41.7)	5	53.5	(46.1. 62.2)
O6-BG+IRN	5	100.5	(83.9, 120.3)	5	121.5	(108.9, 134.3)	4	215.3	(131.6, 352.3)
TMZ+IRN	10	125.2	(109.9, 144)	11	122.7	(113.3, 131.6)	5	181.3	(121.5, 270.4)
Triple	5	186.2	(119.8, 289.6)	6	173.5	(132.8, 226.8)	5	361.4	(198.1, 659.4)

Abbreviations: IRN=irinotecan; O6-BG=O^6^-benzylguanine; TMZ=temozolomide.

a Number of mice evaluated.

b Geometric average day for tumours to reach 1500 mm^3^. The mean survival days and 95% confidence intervals take into account the censored mice (those that were alive at the last follow-up date and did not have tumour equal or above 1500 mm^3^). The maximum likelihood analysis was used to perform calculations (http://www.weibull.com/LifeDataWeb/analysis_parameter_methods.htm).

c Confidence intervals.

d Experiments for each xenograft line were split in two parts conducted in short succession (1–2 months apart) and, therefore, two sets of the control mice were used for each xenograft. The combination of temozolomide and irinotecan was tested twice in SMS-KCNR and CHLA-136 xenografts to confirm the reproducibility of our findings in the models.
